# Heat-Labile Enterotoxin Decreases Macrophage Phagocytosis of Enterotoxigenic *Escherichia coli*

**DOI:** 10.3390/microorganisms11082121

**Published:** 2023-08-21

**Authors:** Ian E. Hollifield, Natalya I. Motyka, Kaylynn A. Fernando, Jacob P. Bitoun

**Affiliations:** Department of Microbiology and Immunology, Tulane University School of Medicine, 1430 Tulane Avenue, #8638, New Orleans, LA 70112, USA; ihollifield@tulane.edu (I.E.H.); nmotyka@tulane.edu (N.I.M.); kfernando@tulane.edu (K.A.F.)

**Keywords:** enterotoxins, diarrhea, ETEC, macrophages, colonization factors, mucosal and systemic ETEC immunity

## Abstract

Enterotoxigenic *E. coli* (ETEC) are endemic in low-resource settings and cause robust secretory diarrheal disease in children less than five years of age. ETEC cause secretory diarrhea by producing the heat-stable (ST) and/or heat-labile (LT) enterotoxins. Recent studies have shown that ETEC can be carried asymptomatically in children and adults, but how ETEC subvert mucosal immunity to establish intestinal residency remains unclear. Macrophages are innate immune cells that can be exploited by enteric pathogens to evade mucosal immunity, so we interrogated the ability of ETEC and other *E. coli* pathovars to survive within macrophages. Using gentamicin protection assays, we show that ETEC H10407 is phagocytosed more readily than other ETEC and non-ETEC isolates. Furthermore, we demonstrate that ETEC H10407, at high bacterial burdens, causes nitrite accumulation in macrophages, which is indicative of a proinflammatory macrophage nitric oxide killing response. However, at low bacterial burdens, ETEC H10407 remains viable within macrophages for an extended period without nitrite accumulation. We demonstrate that LT, but not ST, intoxication decreases the number of ETEC phagocytosed by macrophages. Furthermore, we now show that macrophages exposed simultaneously to LPS and LT produce IL-33, which is a cytokine implicated in promoting macrophage alternative activation, iron recycling, and intestinal repair. Lastly, iron restriction using deferoxamine induces IL-33 receptor (IL-33R) expression and allows ETEC to escape macrophages. Altogether, these data demonstrate that LT provides ETEC with the ability to decrease the perceived ETEC burden and suppresses the initiation of inflammation. Furthermore, these data suggest that host IL-33/IL-33R signaling may augment pathways that promote iron restriction to facilitate ETEC escape from macrophages. These data could help explain novel mechanisms of immune subversion that may contribute to asymptomatic ETEC carriage.

## 1. Introduction

Enterotoxigenic *Escherichia coli* (ETEC) are prominent causes of diarrheal disease that particularly affect children in low-to-middle income countries [1]. Asymptomatic ETEC carriage has been reported in both children and adults [2,3,4], but the sequence of events leading to symptomatic infection or asymptomatic carriage remains unknown. Furthermore, sustained exposure to ETEC or other enteropathogens predisposes children to other co-morbidities including growth faltering [5,6], childhood anemia [7], or intellectual stunting [6,8]. Although the precise molecular mechanisms of ETEC pathogenesis that cause these co-morbidities have not been elucidated, they are likely an outcome following repeated exposure from the heat-stable (ST) and/or heat-labile (LT) enterotoxins.

LT and ST cause secretory diarrhea by increasing cAMP and cGMP second messenger signaling, respectively, and activation of the cystic fibrosis transmembrane receptor results in water and electrolyte loss [9]. ETEC are classically considered non-invasive and non-inflammatory, but sometimes, ETEC can cause low-grade fevers in symptomatic individuals [10]. In some cases, ETEC can be disseminated into extraintestinal tissues including the mesenteric lymph nodes, spleen, and liver [11] without overt inflammation, but how they are trafficked to these tissues and whether these sites represent a potential source for reinoculation of the intestinal mucosa following primary infection remains unresolved. ETEC may compromise intestinal barrier integrity [12], or they may be trafficked to non-intestinal sites via immune cells [11]. Intranasal ETEC infection models have demonstrated that macrophages are recruited to the mucosae [13]. Furthermore, in vitro co-culture experiments have shown that macrophages can project through intestinal epithelial cell monolayers to phagocytose ETEC and other *E. coli* pathovars [14,15], but macrophages have not received major attention as ETEC reservoirs. Thus, it is important to further understand whether ETEC and its virulence-determining enterotoxins affect macrophage-mediated phagocytosis such that ETEC could subvert mucosal immunity.

Intestinal macrophages represent a heterogenous population of cells originating from either the self-renewal of embryonically seeded yolk sac progenitors or the differentiation of monocytes of hematopoietic origin [16]. Macrophages can be activated by pathogen-associated molecular patterns (PAMPS) including the TLR4 ligand LPS to drive proinflammatory M1 phenotypes, consisting of nitric oxide and proinflammatory cytokine (e.g., TNFα and IL-1β) production [17]. During early infection, M1 macrophages help control the invading pathogen burden, but their secreted factors also lead to intestinal epithelial transcriptional changes that may affect ETEC attachment [18]. However, sustained M1 macrophage activity can lead to inflammation-associated tissue damage, and IL-4 and IL-33 are key cytokines that promote the resolution of inflammation via the conversion of macrophages to the M2 phenotype (e.g., alternative activation) [19]. 

We recently demonstrated that LT and ST stimulate IL-33 production in the murine small intestinal mucosa [20,21]. IL-33 is generally believed to polarize toward type 2 immunity characterized by the induction of the IL-5 and IL-13 cytokines that function to reinforce the intestinal mucosa [22,23]. Although IL-33 is predominantly produced by epithelial cells, some macrophages can produce it after stimulation with a TLR4 signal (e.g., LPS) and a cAMP-elevating agent (e.g., forskolin or adenylate cyclase toxin) [24,25]. We previously demonstrated that LT suppresses IL-33-mediated TNFα production in bone-marrow dendritic cells (BMDCs) and bone marrow macrophages (BMMs) [21]. However, IL-33-stimulated responses developed much more rapidly in BMDCs as compared to BMMs, indicating that signaling through the IL-33 receptor (IL-33R, aka ST2) on macrophages may be dependent on the conditions that elevate its expression [21]. 

In macrophages, IL-33 promotes the M2 phenotype via mitochondrial redox-related mitophagy [26], a process that liberates iron, which is strictly controlled as a well-documented enteropathogen virulence factor. Interestingly, IL-33 and hemin are also required to drive the differentiation of iron-recycling macrophages from circulating monocytes [27], which suggests that IL-33 could initiate the host response to prevent ETEC from accessing iron. Similar to IL-33, the acute iron deprivation of macrophages reprograms cellular metabolism to promote increased citric acid cycle intermediates including itaconate and succinate, which promote M2 phenotypes [28].

Together, these observations led us to question whether ETEC enterotoxins alter IL-33/IL-33R signaling to affect iron homeostasis and promote ETEC escape from macrophages that could lead to asymptomatic carriage. Furthermore, we set out to understand whether macrophages could produce IL-33 following exposure to ETEC (e.g., bearing LPS) and LT (e.g., cAMP-inducing source). Finally, we set out to demonstrate that iron availability is a virulence factor that contributes to ETEC escape from macrophages.

## 2. Materials and Methods

### 2.1. Bacterial Strains and Culture Conditions

ETEC H10407 (CFA/I^+^, STh^+^, STp^+^, LT^+^), ETEC 214-4 (CS6^+^, STp^+^), ETEC B41 (F41^+^, STp^+^), ETEC E24377A (CS1^+^, CS3^+^, STh^+^, LT^+^), ETEC LSN03-016011/A (CS17^+^, LT^+^), adherent and invasive *E. coli* (AIEC) NRG857c, enteroaggregative *E. coli* (EAEC) C3493, enteropathogenic *E. coli* (EPEC) O127:H6, and enterohemorrhagic *E. coli* (EHEC) O157:H7 were streaked from glycerol stocks onto LB agar plates. Isolated colonies were used to inoculate LB overnight cultures before being subcultured in LB broth the next day at a 1:100 dilution. Stationary phase bacterial cultures were harvested via centrifugation for 5 min at 14,000 rpm and then resuspended in sterile PBS for infection in gentamicin protection assays. For multiplicity of infection (MOI) experiments, MOI = 1 corresponds to 5 × 10^5^ bacterial cells per well, MOI = 5 corresponds to 2.5 × 10^6^ bacterial cells per well, and MOI = 10 corresponds to 5 × 10^6^ bacterial cells per well.

### 2.2. Enterotoxins

ST was purified from the supernatants of recombinant strain 9115 as described previously [29]. LT-holotoxin was purified as previously described [20]. ST and LT were used at 1.0 µg per well.

### 2.3. Macrophage Culture

Raw 264.7 murine macrophages were obtained from ATCC and cultured in Dulbecco’s modified Eagle’s medium (DMEM) supplemented with 10% fetal bovine serum (FBS) in tissue culture flasks. Raw cells were harvested via trypsinization and subcultured at 5 × 10^5^ cells per well in 24-well plates for 24 h to allow attachment overnight before experimentation. 

For BMM preparation, femurs and tibias were removed from euthanized female CD1 mice and flushed with sterile PBS through a 70 µm filter. The cells were centrifuged for 5 min at 4 °C and 500× *g* and resuspended in 28 mL complete DMEM (cDMEM) (10% FBS, 5% horse serum, 1% HEPES, 1% sodium pyruvate, 1% GlutaMAX, 1% penicillin–streptomycin). Then, L929 conditioned media (7 mL) containing murine CSF was prepared and added to the cDMEM, as described previously [21]. Macrophage differentiation was carried out for 12 days before experimentation.

Macrophages (Raw 264.7 or BMMs) were then treated with purified LT, purified ST, *Salmonella minnesota* LPS (InvivoGen, cat# R595, San Diego, CA, USA), IL-33 (R&D Systems, cat# 3626-ML, Minneapolis, MN, USA), forskolin (Sigma, cat#F3917, Waltham, MA, USA), CFA/I (BEI Repository, cat# NR-49110, Manassas, VA, USA), or deferoxamine mesylate (Sigma, cat# D9533).

### 2.4. Gentamicin Protection Assays

Raw 264.7 macrophages or BMMs were plated in DMEM supplemented with 10% FBS at 5 × 10^5^ cells per well in 24-well plates for 24 h to allow attachment. The following day, wells were washed 3 times with PBS, and the media were replaced with antibiotic-free DMEM supplemented with 20% FBS. 

Raw 264.7 macrophages or BMMs were infected with ETEC H10407, ETEC 214-4, ETEC B41, ETEC E24377A, ETEC LSN03-016011/A, AIEC NRG857c, EAEC C3493, EPEC O127:H6, and EHEC O157:H7 at MOI of 1, 5, or 10 for 1, 4, 8, or 24 h at 37 °C. Then, the media was removed and replaced with DMEM with 100 µM gentamicin (Gibco, cat# 15710-064, Waltham, MA, USA) to kill non-phagocytosed bacteria. Gentamicin treatment was carried out for 1, 4, 8, or 24 h. Macrophages were osmotically lysed with 1% Triton-X (Sigma-Aldrich, cat# 9002-93-1) to release phagocytosed bacteria, and lysates were plated on LB agar and grown statically overnight at 37 °C. Colony-forming units (CFUs) were enumerated from LB agar plates and represent phagocytosed bacteria that remained viable at each timepoint.

### 2.5. Griess Assay

Nitrite levels were assessed as a proxy for macrophage nitric oxide production by assessing culture supernatant nitrite buildup at noted timepoints with Griess reagent (Invitrogen, cat# G7921, Waltham, MA, USA).

### 2.6. Lactate Dehydrogenase Activity

Macrophage viability was assessed via measurement of lactate dehydrogenase (LDH) activity in cell culture supernatants using the LDH cytotoxicity assay kit (Thermo, cat# 88953, Waltham, MA, USA). Data are reported as percent lysis by taking the ratio of the absorbance (490 nm) of cell culture supernatants to the absorbance (490 nm) of lysed untreated control cells.

### 2.7. ELISA

Cell culture lysates or supernatants were collected, clarified, and their protein content was quantified through the bicinchoninic acid (BCA) assay (Thermo Fisher, cat no. 23225). These samples were then applied to IL-33 (R&D Systems, #DY3626) or TNFα (R&D Systems, #DY410) DuoSet ELISAs according to the manufacturer’s instructions. Experiments were repeated at least three times. Cytokine measurements are calibrated per microgram of total protein for cell lysates but represented as nanograms per milliliter for macrophage secretions.

### 2.8. Flow Cytometry

Raw 264.7 macrophages were treated with IL-33 (1–100 ng) or deferoxamine mesylate (0.1–1.5 mM) for 24 h before being trypsinized and counted. Cells were then washed with 500 µL cell sorting buffer (CSB; 1× PBS, 2% newborn calf serum, 0.1% NaN_3_, 5 mM EDTA, pH 8.0) and blocked for 15 min with an Fc block derived from a 2.4G2 hybridoma cell (ATCC, HB-197) supernatant. PerCP/Cy5.5 CD80 (BioLegend, cat# 104722, San Diego, CA, USA), PE-CD86 (BioLegend, cat# 159204), APC-CD40 (BioLegend, cat# 124612), PE-Cy7-IA/IE (BioLegend, cat# 107630), FITC IL-33R (MD Bioproducts, cat# 101001F, St. Paul, MN, USA), and aqua fluorescent live/dead stain (Invitrogen, cat# L34966) were added directly to cells following Fc block incubation for 30 min at 4 °C protected from light. Then, the cells were washed with CSB and fixed for 20 min at room temperature in 2% paraformaldehyde protected from light. The cells were washed again with CSB and resuspended in 500 µL fresh CSB for analysis via flow cytometry on a BD LSR Fortessa.

### 2.9. Statistical Analysis

All statistical analyses were performed using Prism 9 software (GraphPad, Inc., San Diego, CA, USA). Unpaired student’s *t*-tests were used to analyze experiments containing two groups. In experiments with more than two groups, statistical analysis was performed using an unpaired one-way analysis of variance (ANOVA) followed by post hoc analysis for multiple comparisons, as described in the figure legends. Experiments monitoring multiple treatment groups at different timepoints were analyzed with two-way ANOVA followed by post hoc analysis as described in the figure legends. Biological significance is defined as 5% or less chance (*p* < 0.05) that results were obtained due to chance.

## 3. Results

### 3.1. Macrophages Phagocytose ETEC

**Figure 1:**
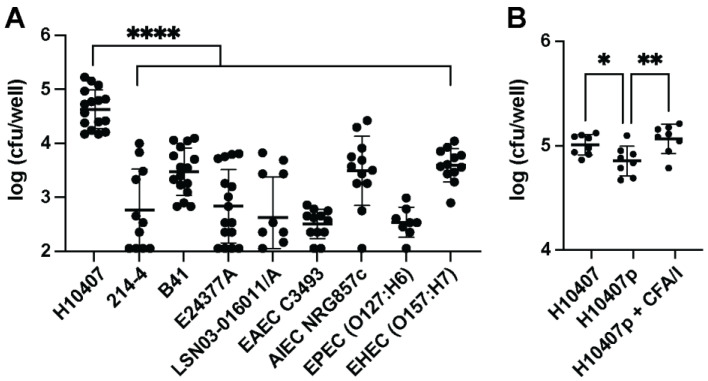
Recoverable intracellular bacterial load of ETEC H10407 in macrophages differs from other ETEC and *E. coli* pathovars. Gentamicin protection assays performed on ETEC H10407 compared to other *E. coli* pathovars or other ETEC isolates revealed greater numbers of viable H10407 inside macrophages. Raw 264.7 macrophages (5 × 10^5^ cells) were added to the wells of 24-well cell culture plates and allowed to attach for 24 h prior to infection. Bacteria were added at an MOI = 1 and allowed to incubate with cells 1 h in DMEM with 20% FBS. After incubation, wells were washed, and antibiotic-free media were replaced with gentamicin-containing media for the remainder of the assay. When macrophages are infected with equal numbers of H10407 compared to a selection of other *E. coli* isolates, recovered intracellular H10407 CFUs at one-hour post-infection were significantly greater than those recovered from the other isolates assessed (**A**). H10407 was infected in equal numbers as H10407P, which is an H10407 variant that has lost the CFA/I-containing virulence plasmid (p948). H10407 was recovered from macrophage lysates in greater numbers than H10407p, but the addition of CFA/I (1 µg) rescued the ability of H10407p to be phagocytosed (**B**). Data in (**A**,**B**) have been aggregated from three individual experiments containing 2–4 replicates per experiment. Error bars represent the standard deviation of log transformed data. Each dot represents a single well following a gentamicin protection assay. Data in (**A**,**B**) were analyzed in Prism 9 via one-way ANOVA with Dunnett’s test for multiple comparisons, ****, *p* < 0.0001; **, *p* < 0.05, *, *p* < 0.05.

We began our investigation by performing gentamicin protection assays using Raw 264.7 macrophages or BMMs and gentamicin-sensitive ETEC strains and other pathogenic, but non-enterotoxigenic, *E. coli* strains. Previous studies have demonstrated that adherent and invasive *E. coli* (AIEC) can survive within the phagolysosome of macrophages via the induction of an acid and stringent response [30], but we are unaware of studies that have compared macrophage-mediated phagocytosis of ETEC strains with AIEC, EAEC, EPEC, and EHEC. We chose to assess macrophage-mediated phagocytosis of ETEC strains that have been assessed in in human challenge studies—H10407 [31], 214-4 [32], E24377A [33], and LSN03-016011/A [34]—or caused disease in farm animals, B41 [35]. These clinical ETEC isolates represent the diversity of wild ETEC with different colonization factor and enterotoxin profiles. We infected Raw 264.7 macrophages with these ETEC strains and EAEC C3493, AIEC NRG857c, EPEC (O127:H6), and EHEC (O157:H7) at the MOI of 1 for 1 h before osmotic lysis to release intraphagosomal bacteria. We chose the MOI of 1 (5 × 10^5^ CFU to 5 × 10^5^ macrophages) to represent a low-dose infection. As expected, our results demonstrate that macrophages phagocytose all *E. coli* assayed, but surprisingly, ETEC H10407 is phagocytosed at a significantly (*p* < 0.0001) higher rate than other ETEC and non-ETEC isolates (Figure 1A). ETEC H10407 represents one of the most diarrheagenic and burdensome ETEC isolates, so these data suggest that macrophages may be important innate immune ETEC sensors. ETEC H10407p is a non-diarrheagenic derivative ETEC H10407 [36] that lost the 948 kilobase pair virulence plasmid (p948), which encodes the genes for CFA/I fimbriae [37] among other genes. Since ETEC H10407 was phagocytosed at significantly higher rates than other ETEC and non-ETEC isolates, we compared ETEC H10407 and ETEC H10407p in side-by-side gentamicin protection assays. We show that ETEC H10407 is phagocytosed more efficiently than ETEC H10407p (Figure 1B), although the differences are minor compared to other *E. coli* (Figure 1A). Furthermore, we demonstrate that the addition of exogenous CFA/I to ETEC H10407p rescues macrophage-mediated phagocytosis to the level observed using ETEC H10407. Colonization factors may promote macrophage-mediated phagocytosis, but it also remains possible that other factors contribute to these phenotypes.

### 3.2. LT Intoxication of Macrophages before ETEC Infection Decreases Recoverable ETEC Burden 

**Figure 2:**
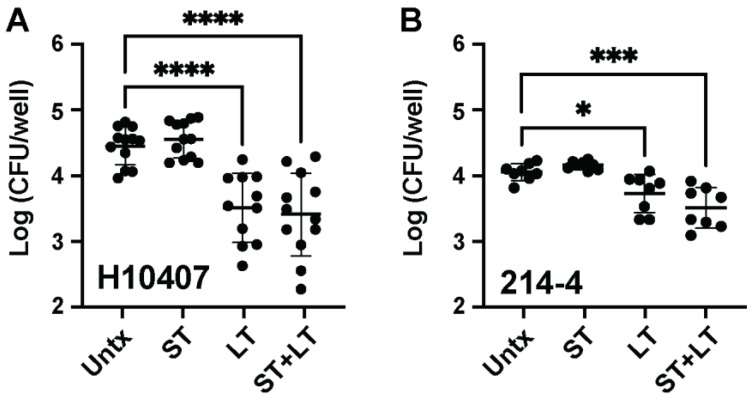
Pretreatment of macrophages with LT, but not ST, decreases phagocytosis of ETEC H10407 and ETEC 214-4. Raw 264.7 macrophages (5 × 10^5^ cells) were plated in 24-well cell culture plates as above. Cells were pretreated for 24 h with 2 µg/mL (1 µg/well) of the noted toxins; then, a gentamicin protection assay was performed as above. Pretreated macrophages were infected with H10407 (**A**) or 214-4 (**B**) at an MOI = 1. Pretreatment with LT alone and LT and ST together, but not ST alone, decreased recovered intracellular bacteria when infected with either isolate. Data in (**A**,**B**) have been aggregated from three individual experiments containing 2–4 replicates per experiment. Error bars represent the standard deviation of log transformed data. Each dot represents a single well following a gentamicin protection assay. Data in (**A**,**B**) were analyzed in Prism 9 via one-way ANOVA with Dunnett’s test for multiple comparisons. *, *p* < 0.05; ***, *p* < 0.001; ****, *p* < 0.0001.

LT intoxication suppresses TNFα proinflammatory cytokine production following TLR4 and IL-1 receptor stimulation in macrophages [21]. Thus, it would be reasonable to expect that LT intoxication also suppresses the ability of macrophages to phagocytose ETEC. To understand the effect of ETEC enterotoxins on macrophage-mediated ETEC phagocytosis, we pretreated macrophages with ETEC enterotoxins, ST, LT, and ST + LT (1 µg each) for 24 h before performing 1 h gentamicin protection assays following ETEC H10407 or ETEC 214-4 inoculation at an MOI of 1. We used ETEC H10407 and ETEC 214-4 as an LT-ETEC strain and non-LT-ETEC strain, respectively. We show that exogenous LT intoxication significantly suppresses the ability of Raw 264.7 macrophages to phagocytose both ETEC H10407 (*p* < 0.0001) (Figure 2A) and ETEC 214-4 (*p* < 0.05) (Figure 2B). We also demonstrate that LT intoxication suppresses the ability of BMMs to phagocytose ETEC H10407 (Supplemental Figure S1). On the other hand, ST does not affect the ability of macrophages to phagocytose ETEC H10407 (Figure 2A) or ETEC 214-4 (Figure 2B). This is expected, since immune cells predominantly do not express the ST receptor, guanylyl cyclase C. As expected and similar to the results obtained following LT intoxication, simultaneous ST + LT intoxication significantly suppresses the ability of macrophages to phagocytose ETEC H10407 (*p* < 0.0001) (Figure 2A) and ETEC 214-4 (*p* < 0.001) (Figure 2B). These data demonstrate that LT intoxication significantly decreases the ability of macrophages to phagocytose ETEC, particularly ETEC H10407. This suggests that LT may function to decrease the perceived infectious dose of ETEC, which may be important for ETEC strains that are robustly phagocytosed like ETEC H10407. 

### 3.3. ETEC Can Persist Inside Macrophages

**Figure 3:**
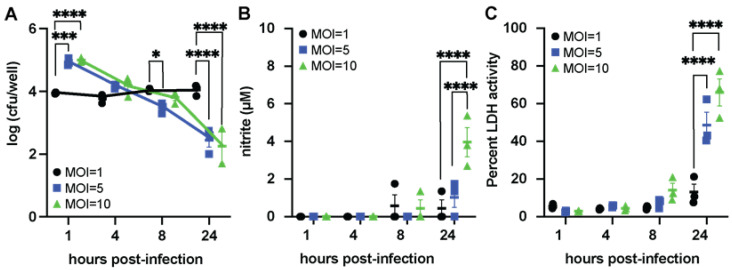
Lower infectious doses of H10407 show stable persistence within macrophages without inflammation. Macrophages incubated with ETEC H10407 at an MOI = 1 showed increased CFUs of intracellular ETEC H10407 at 8 and 24 h post-infection, while macrophages incubated with ETEC H10407 at an MOI = 5 or MOI = 10 showed reduced CFUs of intracellular ETEC H10407 at 8 and 24 h post-infection (**A**). Nitrite levels, a proxy measurement for nitric oxide production, were higher in the macrophages infected at MOI = 5 or MOI = 10 than in the MOI = 1 group (**B**). LDH activity was also elevated in the supernatants of the cells infected at higher MOIs at 24 h (**C**). Data are representative of three individual experiments containing 3 replicates per experiment. Each dot represents a single well following a gentamicin protection assay. Data were analyzed via two-way ANOVA with timepoints matched with Sidak’s test for multiple comparisons. *, *p* < 0.05; ***, *p* < 0.001; ****, *p* < 0.0001.

Since LT suppresses the ability of macrophages to phagocytose ETEC at an MOI = 1, we assessed whether the dose of ETEC H10407 affected phagocytosis and macrophage-mediated inflammation. We characterized two markers of inflammation, nitrite (as a proxy for bactericidal NO production) and LDH (as a proxy for tissue damage) in ETEC-H10407 infected macrophage cultures. For this experiment, Raw 264.7 macrophages were treated with ETEC H10407 at an MOI of 1, 5, or 10 and then were assessed in gentamicin protection assays kinetically for 1, 4, 8, and 24 h post-infection, as shown in Figure 3. Our data demonstrate that the severity of ETEC infection (e.g., dose or MOI) may determine whether macrophages mount inflammatory responses against ETEC. Surprisingly, we found that the CFUs of intracellular ETEC H10407 recovered from macrophages infected at an MOI of 1 were not significantly different at 1, 4, 8, and 24 h post-infection in gentamicin-containing media (Figure 3A, black circles). On the other hand, at an MOI of 5 or 10, macrophages begin to kill ETEC H10407, as shown by the decrease in intracellular CFUs 4, 8, and 24 h post-infection (Figure 3A, MOI = 5, blue squares; MOI = 10, green triangles). We show that macrophages infected with ETEC H10407 for 24 h at an MOI of 10 have significantly more nitrite than macrophages infected with ETEC H10407 for 24 h at MOI values of 1.0 and 5.0 (Figure 3B). This suggests that macrophages are initiating NO-mediated killing, as has been demonstrated for other enteric pathogens [38]. Furthermore, we demonstrate that macrophages infected with ETEC H10407 for 24 h at an MOI of 5 or 10 have significantly higher LDH activity than macrophages infected with ETEC H10407 for 24 h at an MOI of 1 (Figure 3C). We also show that macrophages permit the persistence of ETEC 214-4 when inoculated at an MOI of 1, but macrophages begin to kill ETEC 214-4 when inoculated at an MOI of 5 or 10 (Supplemental Figure S2). These data suggest that at low infecting doses, ETEC are phagocytosed by macrophages but escape proinflammatory killing responses. 

### 3.4. LT and LPS Promote Macrophage IL-33 Production

**Figure 4:**
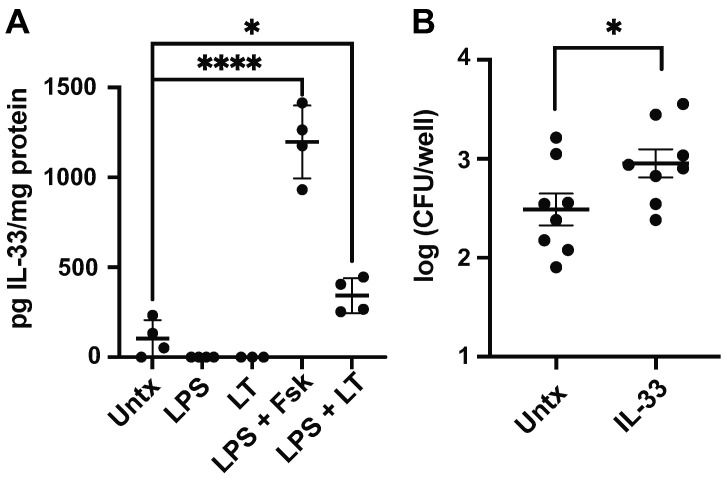
Simultaneous LT and LPS treatment induces IL-33 in macrophages. BMMs were treated with LPS (50 ng), LT (1.0 µg), LPS + cAMP-inducer forskolin (Fsk, 20 µM), or LPS and LT, and compared to untreated (untx) controls for IL-33 production. Neither LPS nor LT alone is sufficient to induce IL-33 alone in BMMs, but the simultaneous exposure of BMMs with LPS + LT causes increased IL-33 production, as detected in BMM lysates 8 h following exposure. LPS + Fsk is the positive control. BMMs were stimulated with or without IL-33 (10 ng) for five days before ETEC H10407 infection at an MOI of 1 in gentamicin protection assays for 1 h (**B**). IL-33 pretreatment promotes enhanced ETEC H10407 survival within BMMs. Data in (**A**,**B**) are representative of three independent experiments containing 3–4 replicates per experiment. Each dot represents a single well containing BMMs. Data in (**A**) were analyzed via one-way ANOVA with Dunnett’s test for multiple comparisons, *, *p* < 0.05; ****, *p* < 0.0001 and data in (**B**) were analyzed via Student’s *t*-test, *, *p* < 0.05.

Since LT functions in suppressing otherwise proinflammatory responses in macrophages, it remains possible that LT polarizes macrophages to the M2 phenotype that allows for extended intracellular pathogen survival [39]. IL-33 is a mucosal cytokine that promotes M2 polarization and previous studies have demonstrated that BMMs stimulated with LPS and a cAMP-inducing agent (e.g., forskolin or adenylate cyclase toxin) produce IL-33 [40]. As LT acts through cAMP induction, we sought to confirm that it similarly induces macrophage IL-33 in concert with LPS stimulation. Our data demonstrate that LT and LPS synergize to produce IL-33 in BMMs (Figure 4A). As shown using 1 h gentamicin protection assays, IL-33-stimulated BMMs are significantly more permissive of ETEC H10407 infection (Figure 4B). It is important to note that IL-33 was not secreted but found in BMM lysates. Earlier, we demonstrated that the induction of the proinflammatory M1-like macrophage phenotype, characterized by secreted TNFα and nitrite production, is dependent on perceived ETEC burden. It is possible that LT-mediated IL-33 production in macrophages likely stimulates an IL-4-independent phenotypic conversion toward an M2 phenotype characterized by increased aconitate and succinate [28].

### 3.5. Iron Limitation Decreases Macrophage Response to ETEC

**Figure 5:**
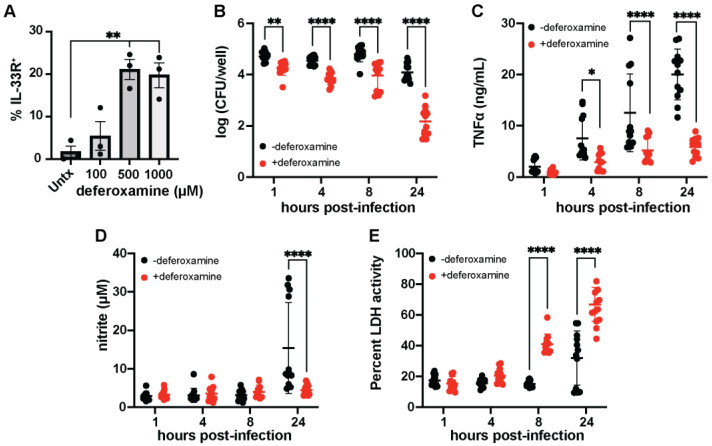
Iron restriction via deferoxamine induces IL−33R and promotes ETEC escape from macrophages. Raw 264.7 macrophages (5 × 10^5^ cells) were added to each well of a 24-well cell culture plate and treated with deferoxamine mesylate (1 mg/mL) for 24 h before the addition of ETEC H10407 at MOI = 1. Deferoxamine induces the expression of IL-33R on macrophages (**A**). Macrophages were allowed to phagocytose ETEC H10407 for 1 h in DMEM containing 20% FBS. Then, the wells were washed with PBS, and gentamicin-containing DMEM was added to kill non-phagocytosed ETEC. Intracellular CFUs were recovered following 1, 4, 8, and 24 h by osmotically lysing macrophages. Deferoxamine significantly suppresses macrophage-mediated ETEC H10407 phagocytosis (**B**). Deferoxamine significantly suppresses macrophage TNFα secretion 4, 8, and 24 h post ETEC H10407 infection (**C**). Deferoxamine significantly suppresses the macrophage production of nitrite 24 h post-ETEC H10407 infection (**D**). Deferoxamine significantly increases macrophage LDU activity 8 and 24 h post-ETEC H10407 infection (**E**). Data have been aggregated from three individual experiments containing 2–4 replicates per experiment. Data were analyzed via two-way ANOVA with timepoints matched with Sidak’s test for multiple comparisons. *, *p* < 0.05; **, *p* < 0.01; ****, *p* < 0.0001.

Iron is required for the efficient macrophage killing of enteric pathogens [38], and IL-33 has been previously associated with the differentiation of iron recycling macrophages [27]. We next investigated whether iron restriction alters the IL-33/IL-33R signaling of macrophages. We initially observed that Raw 264.7 macrophages can respond to IL-33 stimulation, and IL-33 enhances CD80, CD86, CD40, and IA/IE expression (Supplemental Figure S3). Therefore, we treated macrophages with the siderophore deferoxamine mesylate and measured IL-33R expression using flow cytometry. We show that deferoxamine significantly induces IL-33R expression on macrophages (Figure 5A), suggesting that iron restriction promotes alternative activation. Since macrophages are the principal cells responsible for both iron recycling and limiting free iron in response to infection [27], we wondered how iron limitation would affect macrophage-mediated ETEC killing. Using gentamicin protection assays to assess intracellular bacterial burdens, we tested whether 24 h deferoxamine pretreatment altered the macrophage phagocytosis of ETEC H10407 in vitro. We found decreased intracellular ETEC H10407 in the deferoxamine-treated macrophages as compared to control macrophages, following 1 h gentamicin treatment, but extending gentamicin treatment to 4, 8, and 24 h significantly decreases viable intracellular ETEC H10407 recovered from macrophage lysates (Figure 5B). It is important to note that deferoxamine was removed from the cultures before gentamicin was added, so the decreased ETEC H10407 burden in deferoxamine-treated macrophages noted following 24 h gentamicin treatment likely reflects ETEC H10407 escape from macrophages and gentamicin-mediated killing. In support, we demonstrate that deferoxamine treatment significantly decreases the ability of macrophages to secrete TNFα following 4, 8, and 24 h gentamicin treatment. (Figure 5C). This suggests that deferoxamine treatment polarizes away from proinflammatory M1 phenotypes. We also demonstrate that the deferoxamine-treated macrophages have significantly less nitrite after 24 h gentamicin treatment as compared to untreated but ETEC H10407-infected macrophages (Figure 5D). This demonstrates that they are not able to promote oxidative responses that lead to bacterial killing. Furthermore, deferoxamine-treated macrophages have increased LDH activity (Figure 5E) indicative of increased damage. It is important to note that macrophage viability was similar between deferoxamine-treated and untreated groups following 1 h gentamicin treatment. This interpretation accounts for the differences in viability not appearing until 8 h post-infection over 9 h after the removal of deferoxamine from the culture. 

## 4. Discussion

Children can be infected with similar strains of ETEC without developing long-term protective immunity [10]. For LT-expressing ETEC infections, LT intoxication should boost the immune response to co-delivered bacterial antigens and protect against subsequent infection. However, long-term asymptomatic ETEC persistence has been documented in patients without mechanisms that explain how ETEC subvert immune responses to establish intestinal residence [2,3,4]. Here, we present a model in which macrophages may be exploited by ETEC via alterations to phagocytosis and macrophage-mediated inflammation. Macrophages are a highly diverse subset of myeloid cells that patrol the intestinal lamina propria, where they reside underneath a layer of epithelial cells and phagocytose ETEC through projections between the epithelial cell junctions [14,15].

Our data show that ETEC H10407, a diarrheagenic and burdensome ETEC isolate, is unique in that it has an elevated ability to be phagocytosed by macrophages. Currently, it is unclear if this ability is a virulence determinant, but it suggests that other wild ETEC isolates may also be preferentially phagocytosed by macrophages. However, we also demonstrate that LT, but not ST, decreases the perceived ETEC burden, potentially by inhibiting macrophage phagocytic activity. LT also polarizes cytokine responses and induces the production of surface-bound activation markers in monocytes, macrophages [21], dendritic cells [41], and mast cells [42]. Therefore, besides diarrhea, LT may suppress or prevent the initiation of otherwise robust proinflammatory responses to ETEC. 

We have previously demonstrated that both ST and LT enterotoxins induce IL-33 production in T84 colonic epithelial cells and in the small intestinal mucosa of intoxicated mice [20,21]. Here, we also demonstrate that the combination of LPS and LT induces IL-33 production in macrophages. Although classically considered a pro-type 2 polarizing cytokine, IL-33 induces the production of reactive oxygen species in human monocytes in vitro [26]. We speculate that enterotoxin-mediated IL-33/IL-33R signaling initiates pathways that lead processes to sequester and restore the mucosal iron balance following enteric infection. Intestinal macrophages require constant replenishment by circulating monocytes, which suggests that enterotoxin-mediated IL-33/IL-33R signaling could promote the differentiation of incoming monocytes, potentially to promote iron recycling via red pulp macrophage phenotypes [27]. IL-33 induces the production of mitochondrial reactive oxygen species that leads to mitophagy and a shift from TNFα and CXCL-10 (e.g., M1 phenotype) production to CCL22 (e.g., M2 phenotype) production in THP1 cells [26]. Furthermore, IL-33 promotes anemia during chronic inflammation by inhibiting the differentiation of erythroid progenitors [43]. Ultimately, ETEC must compete with other microbes and the host for nutrient acquisition, and these data suggest that the prevalent childhood anemia in low- and middle-income countries [7] may also be an emerging co-morbidity of ETEC and other enteropathogens. 

Iron restriction has also been found to reduce the inflammatory response of macrophages to LPS exposure [28,44], and we demonstrate that deferoxamine treatment allows ETEC to escape macrophages. The blockade of Na^+^/H^+^ exchangers (e.g., NHE3) abolishes deferoxamine-mediated IL-8 production [45], and it remains plausible that LT could suppress inflammation by mediating a blockade of intestinal NHE3. Furthermore, the IL-33 stimulation of macrophages results in mitochondrial uncoupling and an increased production of TCA metabolites itaconate, succinate, and fumarate [19], which are known to increase the virulence of enteric pathogens [46]. Recent studies on *V. cholerae* demonstrated that cholera toxin (CT), which is highly homologous to LT, provides CT-expressing *Vibrios* a competitive advantage over non-CT-expressing *Vibrios* for iron and long-chained fatty acid acquisition [47]. While we did not see direct effects of ST on macrophage phagocytosis, we have previously observed that ST causes a depletion of mucosal iron [48], suggesting that the host may carefully control iron availability following ETEC pathogenesis. Furthermore, ST binds iron under anaerobic conditions [29], which suggests that both enterotoxins are involved in pathways that implicate iron as a virulence factor for ETEC. 

It may not be surprising that the outcomes of enterotoxin exposure (e.g., IL-33 production and subsequent IL-33/IL-33R signaling) seek to enhance the host’s ability to withhold iron resources that ultimately prevent ETEC from fully thriving in the intoxicated mucosa. In support, IL-33-stimulated BMMs produce significantly more lipocalin-2 (Lcn2) transcripts, which is an innate protein that obstructs the bacteria’s ability to acquire iron-containing siderophores and limits bacterial growth [49]. 

It is important to further our understanding of macrophages and other innate immune cells during ETEC infection. Here, we provide evidence that ETEC may use their enterotoxins to directly (via cAMP) and indirectly (via IL-33 and iron depletion) promote alternatively activated macrophages. LT provides ETEC with a colonization advantage [50,51], but it also reprograms the mucosal immune response. Our data suggest that LT decreases the perceived ETEC dose, suppresses deleterious inflammatory processes, and promotes ETEC persistence. This suggests that LT may provide an ETEC with a temporary niche that enables their trafficking among non-intestinal tissues [11] where they eventually emerge from to cause repeated symptomatic or asymptomatic infection. 

### Limitations

This study provides evidence that ETEC can evade macrophage-mediated killing. This study is limited by using two different macrophage models in vitro. This study is limited by the lack of in vivo animal data. 

## 5. Conclusions

Although in vitro co-culture experiments have shown that macrophages can project through a layer of intestinal epithelial cells to phagocytose ETEC, little is known about the role of macrophages in ETEC infection. To address this gap in understanding, we first assessed whether ETEC interacts with macrophages in a manner distinct from other *E. coli* pathotypes. Macrophages infected with ETEC H10407 demonstrate a significantly higher burden of intracellular bacteria than those infected with other *E. coli* pathovars. Using gentamicin protection assays, we show that LT intoxication, alone or in combination with ST, reduced macrophage-mediated ETEC phagocytosis. We demonstrate that ETEC may employ LT to lower the perceived ETEC dose to prevent the initiation of inflammatory responses to ETEC. This then allows ETEC to survive within macrophages for some time. The combination of LPS and LT induces IL-33 production in macrophages, and macrophages treated with IL-33 allow increased ETEC persistence. Given the known relationship of macrophages and iron, we show that iron chelation with deferoxamine allows ETEC to escape macrophages. This suggests that macrophages may be able to transport ETEC to different tissues and lead to increased asymptomatic ETEC carriage.

## Data Availability

The data that support the findings of this study are available from the corresponding author upon request.

## Supplementary Materials

The following supporting information can be downloaded at: https://www.mdpi.com/article/10.3390/microorganisms11082121/s1, Figure S1: LT intoxication decreases macrophage-mediated phagocytosis; Figure S2: Kinetics of ETEC 214-4; Figure S3: IL-33 stimulation significantly increases the expression of activation markers.
