# Supplementary material for: Heat-Labile Enterotoxin Decreases Macrophage Phagocytosis of Enterotoxigenic Escherichia coli
Source: Microorganisms. 2023 Aug 21;11(8):2121. doi: 10.3390/microorganisms11082121 (PMC10459231; doi:10.3390/microorganisms11082121)
Supplement: Supplementary file 1 [file microorganisms-11-02121-s001.zip › microorganisms-2509953-supplementary.pdf]

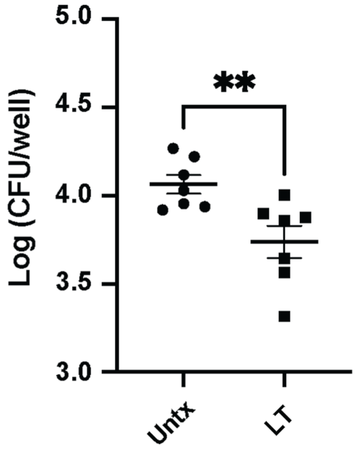

Figure S1. LT intoxication decreases BMM phagocytosis. BMMs (5 × 10^5 cells) were plated in 24-well cell culture plates and pretreated with LT (1µg/well) for 24 hours. Gentamicin protection assays were carried out by inoculating ETEC H10407 at MOI of 1. Data were aggregated from two individiual experiments containing 3-4 replicates per experiment and analyzed via student's *t*-test. **, *p* < 0.01.

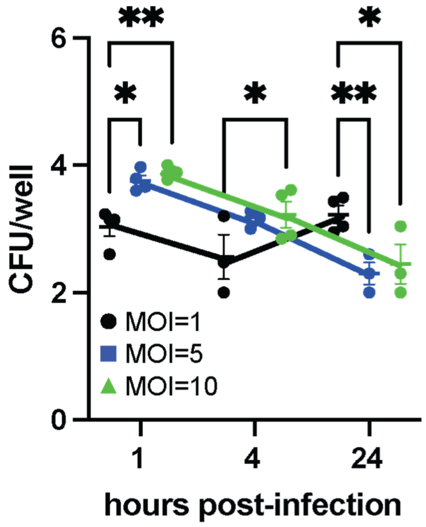

Figure S2. Kinetics of ETEC 214-4 in Raw 264.7 macrophages. Data are representative from one experiment with 3-4 replicates. Data were analyzed via two-way ANOVA with timepoints matched with Šídák's test for multiple comparisons. *, p <0.05; **, p <0.01.

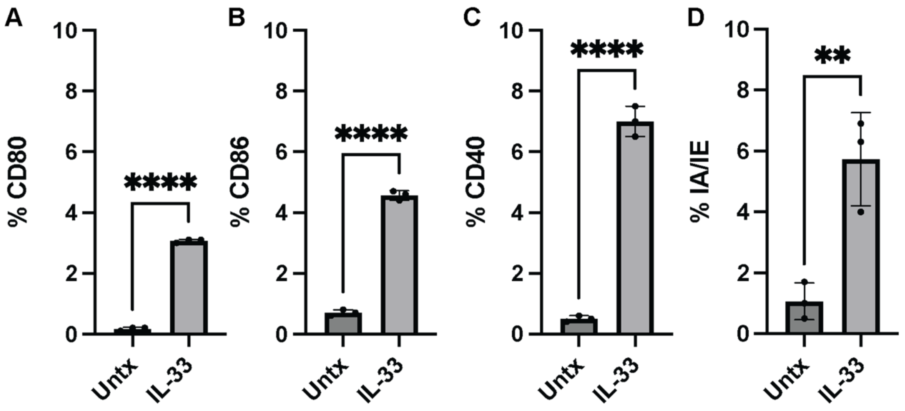

Figure S3. IL-33 stimulation significantly increases the expression of activation markers CD80 **(A)**, CD86 **(B)**, CD40 **(C)**, and IA/IE **(D)** on Raw 264.7 macrophages. Raw 264.7 macrophages were stimulated with IL-33 (10 ng) or left untreated for 24 hours before being analyzed via flow cytometry. Data were aggregated from three individual experiments and statistically analyzed via the student's *t*-test, $p < 0.01$, **, $p < 0.0001$, ****.
